# Clinicopathological Characteristics and Survival Outcomes of Male Breast Lymphoma

**DOI:** 10.3390/jcm15187136

**Published:** 2026-09-14

**Authors:** Abdulmunir Azizy, Izzet Dogan, Mustafa Bozkurt, Onur Dulgeroglu, Serap Yucel, Ibrahim Yildiz, Irmak Durur Subasi, Ali Arican, Cihan Uras

**Affiliations:** 1 Medical Oncology, Istanbul University Institute of Oncology, Istanbul 34093, Turkey; 2 Medical Oncology, Acibadem Healthcare Group, Istanbul 34457, Turkey; dr.izzetdogan@gmail.com (I.D.); mustafa.bozkurt@acibadem.com (M.B.); ibrahim.yildiz@acibadem.edu.tr (I.Y.); ali.arican@acibadem.com (A.A.); 3 General Surgery, Acibadem Healthcare Group, Istanbul 34457, Turkeycihan.uras@acibadem.com (C.U.); 4 Radiation Oncology, Acibadem Healthcare Group, Istanbul 34457, Turkey; serap.yucel1@acibadem.com; 5 Radiology, Acibadem Healthcare Group, Istanbul 34457, Turkey; irmak.durur@acibadem.com

**Keywords:** male breast lymphoma, primary breast lymphoma, SEER, survival

## Abstract

**Background**: Male breast lymphoma is rare, and most evidence on prognosis and treatment comes from female-predominant or mixed cohorts. We evaluated clinicopathological characteristics and cancer-specific survival (CSS) in men with lymphoma recorded as the primary breast site in SEER. **Methods**: Male patients diagnosed between 2000 and 2021 with lymphoma recorded as the primary breast site in SEER were identified. CSS was estimated using Kaplan–Meier methods and compared with the log-rank test. Cox proportional hazards models were used to explore factors associated with CSS. **Results**: A total of 138 male patients were included. Most patients were aged ≥65 years (62.3%). Diffuse large B-cell lymphoma was the most common histological subtype (39.1%), followed by mucosa-associated lymphoid tissue (MALT) lymphoma (23.9%) and follicular lymphoma (17.4%). Among patients with known Ann Arbor stage, stage I disease was the most frequent presentation (48.8%). Chemotherapy was recorded in 44.9% of patients and radiotherapy in 31.2%. In univariable analysis, age (*p* < 0.001), histological subtype (*p* < 0.001), stage (*p* = 0.040), radiotherapy (*p* = 0.038), and chemotherapy (*p* = 0.020) were associated with CSS. Primary breast surgery was not associated with CSS (*p* = 0.556). **Conclusions**: In this SEER-based study of 138 men with breast lymphoma, older age and aggressive histology were independently associated with poorer CSS. Stage and treatment variables showed associations mainly in univariable analysis. These findings should be interpreted cautiously because of missing data, treatment-selection bias, and the lack of detailed treatment information in SEER. This study provides one of the largest and most updated male-only SEER analyses of this rare disease. Larger collaborative datasets with clinical, pathological, molecular, and treatment details are needed to better define prognosis and support individualized treatment.

## 1. Introduction

Breast involvement by lymphoma is rare, and cases in men are particularly uncommon. Because the clinical and imaging findings may resemble breast carcinoma, diagnosis requires tissue biopsy and accurate lymphoma classification [1,2]. The diagnostic criteria proposed by Wiseman and Liao include adequate pathological material, lymphoma in close association with breast tissue, and no disseminated disease at presentation, except possible ipsilateral nodal involvement. Primary breast lymphoma (PBL) occurs mainly in women, but it has also been reported in men. In male patients, the available evidence is mostly limited to case reports and small series [2,3,4].

Patients usually present with a painless unilateral breast mass. Imaging findings are not specific; therefore, histopathological evaluation remains essential. Diffuse large B-cell lymphoma (DLBCL) is the most common subtype, although indolent lymphomas, including Mucosa-associated lymphoid tissue (MALT) lymphoma and follicular lymphoma, may also occur [2,5]. Treatment follows lymphoma-based principles rather than breast cancer treatment pathways. Immunochemotherapy is the main treatment for DLBCL; involved-site radiotherapy may be considered in selected limited-stage cases, and extensive breast surgery is generally avoided when diagnosis and local control can be achieved without surgery [6,7].

In retrospective studies from the rituximab era, immunochemotherapy has been associated with better outcomes in primary breast DLBCL. Radiotherapy may also be useful in selected patients with localized disease [7,8,9]. Age and stage have been reported as important prognostic factors. Some series have also described relapses in the contralateral breast and central nervous system [5,7].

The classification and management of PBL have changed with advances in lymphoma pathology and risk assessment [10]. The World Health Organization and International Consensus classifications now emphasize morphologic evaluation supported by immunophenotypic, molecular, and genetic findings, particularly in aggressive B-cell lymphomas [11]. However, evidence on male breast lymphoma remains very limited because the disease is exceptionally rare and male-specific cohorts are difficult to collect [12]. For this reason, registry-based data can provide important clinical information. A population-based analysis may help clarify the presentation, histologic spectrum, and survival patterns of this rare condition. In this study, we used SEER data to describe clinicopathological characteristics and evaluate factors associated with cancer-specific survival (CSS) among men with breast lymphoma.

## 2. Materials and Methods

This research was conducted as a retrospective cohort analysis. Patient data were extracted from the Surveillance, Epidemiology, and End Results (SEER) database of the National Cancer Institute (NCI), and all records were de-identified prior to analysis. The study was carried out in compliance with the principles of the Declaration of Helsinki and adhered to established good clinical practice standards. We obtained data for this study from the US NCI’s SEER-17 registry [Nov 2023 Sub (2000–2021)]. SEER*Stat program version 9.0.41 was used to extract data. The patient population to be included in the study was determined by selecting from the SEER*Stat program; Site and Mophology Primary Site-labelled = “C50.0-Nipple, C50.1-Central portion of breast, C50.2-Upper-inner quadrant of breast, C50.3-Lower-inner quadrant of breast, C50.4-Upper-outer quadrant of breast, C50.5-Lower-outer quadrant of breast” AND Sex = “Male” AND Behavior code ICD-O-3 = “Malignant” AND ICD-O-3 Hist/behav, malignant = “9590/3: Malignant lymphoma, NOS, 9591/3: Non-Hodgkin lymphoma, NOS, 9596/3: B-cell lymphoma, between diffuse large B and HL (composite HL and NHL), 9597/3: Primary cutaneous follicle centre lymphoma, 9650/3: Classical Hodgkin lymphoma”. Patients were excluded if they were not male, were diagnosed outside the study period, had a non-breast primary site, had non-lymphoma histology, had missing survival information, or were recorded only from death certificate or autopsy reports.

Clinicopathological characteristics such as age, gender, race, origin, tumor location, pathological features, surgical method, and radiotherapy and chemotherapy applications were extracted from the patient group drawn from the database. The causes of death of the patients were recorded. Survival analysis was performed with respect to demographic characteristics and treatment variables. During the assessment of overall survival (OS), the variable “Survival months” was used to define follow-up time, and events were identified as patients classified as “Dead (attributable to this cancer dx)” according to the SEER cause-specific death categorization. Furthermore, both univariable and multivariable analyses were conducted to evaluate the determinants influencing OS.

Baseline variables included age group (<65 vs. ≥65 years), race, laterality, histologic subtype, Ann Arbor stage when available, registry-derived stage group, and B symptoms when recorded. Ann Arbor stage and registry-derived localized, locally advanced, and metastatic groups were analyzed as separate staging variables and were not considered equivalent. The registry-derived stage group was used only for Kaplan–Meier visualization. Treatment variables were obtained from SEER first-course treatment fields and included chemotherapy, radiotherapy, and surgery of the primary site. The breast surgery variable may include different surgical procedures and cannot reliably distinguish diagnostic from therapeutic surgery. The primary endpoint was CSS, defined according to the SEER cause-specific death classification as the time from diagnosis to death attributed to breast lymphoma. Patients alive at last follow-up or dead from other causes were censored.

### Statistical Analysis

Statistical analyses were performed using SPSS version 30.0 (IBM Corp., Armonk, NY, USA) and MedCalc version 23.0.9 (MedCalc Software, Ostend, Belgium). Overall survival was estimated using Kaplan–Meier method, and survival differences between subgroups were compared using log-rank tests. Multivariate analysis was performed using Cox proportional hazarda regression models to identify independent prognostic factors associated with OS. Hazard ratios (HRs) and corresponding 95% confidence intervals (CIs) were calculated. A *p*-value < 0.05 was considered significantly significant.

Variables showing statistical significance in univariable analysis, together with clinically relevant treatment variables, were considered for the multivariable model. Because only 41 cancer-specific death events were observed, the multivariable analysis was limited to variables considered suitable for inclusion. Age, histological subtype, chemotherapy, radiotherapy, and primary breast surgery were included in the multivariable analysis. Stage was not included in the final model because of incomplete stage information, its relationship with treatment variables, and the limited number of events. Missing or unknown values were retained as separate categories in the descriptive analyses. Variables with substantial missing data were not forced into the multivariable model. The proportional hazards assumption was not formally assessed because of the limited number of events and sparse subgroup data.

## 3. Results

Table 1 summarizes baseline characteristics of the 138 male patients. Most patients were aged ≥65 years (62.3%) and White (88.4%). Laterality was similar for right and left breast disease (48.6% vs. 47.1%). DLBCL was the most common histology (39.1%), followed by MALT lymphoma (23.9%) and follicular lymphoma (17.4%). Ann Arbor stage was not recorded in all patients; among patients with known stage, stage I was the largest group.

**Figure 1:**
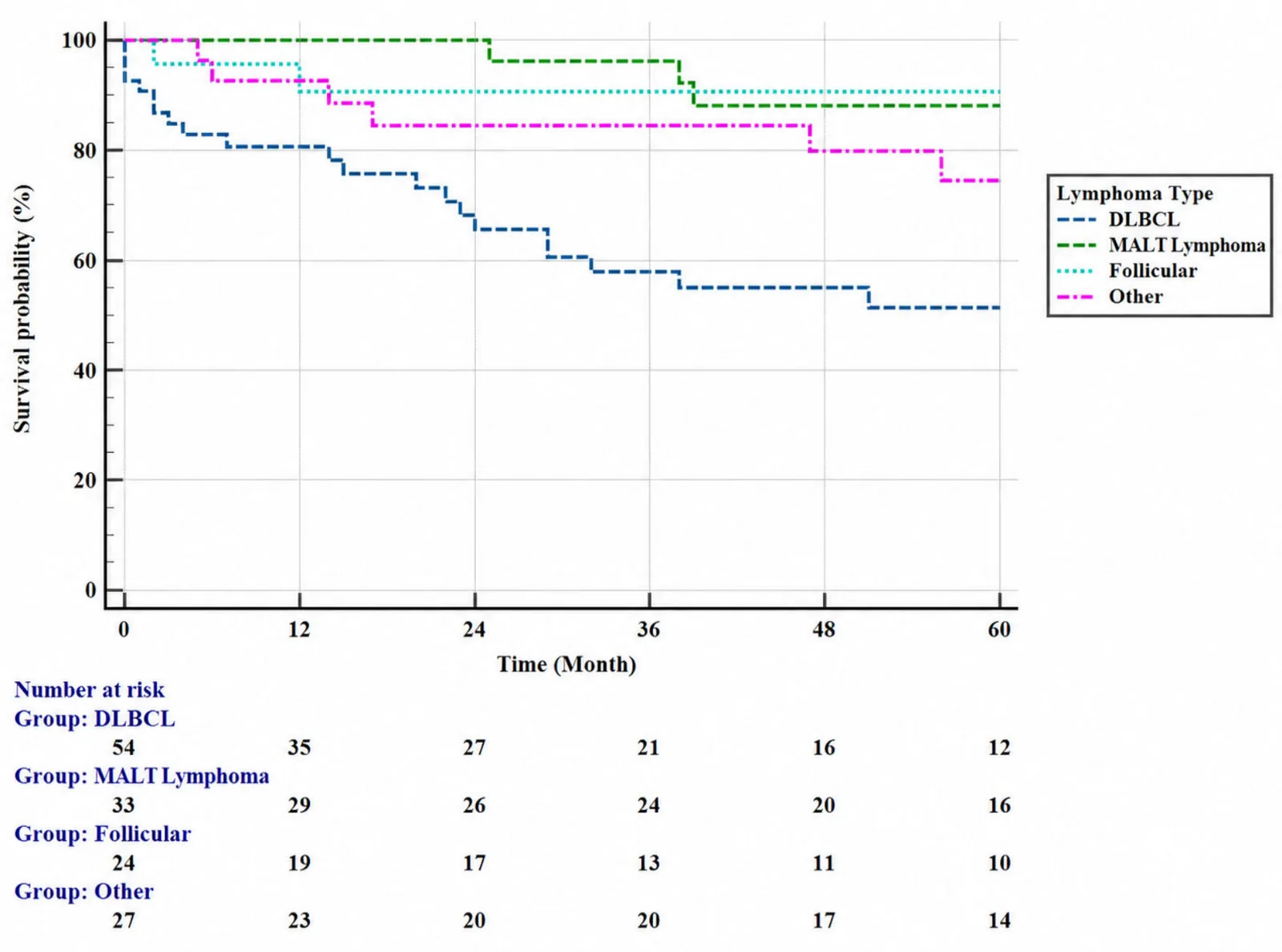
Kaplan–Meier curves of cancer-specific survival stratified by histological subtype (DLBCL, MALT lymphoma, follicular lymphoma, and other).

The histology-stratified Kaplan–Meier curves showed higher CSS in patients with MALT lymphoma and follicular lymphoma than in those with DLBCL. The other lymphoma group showed intermediate survival (Figure 1).

**Figure 2:**
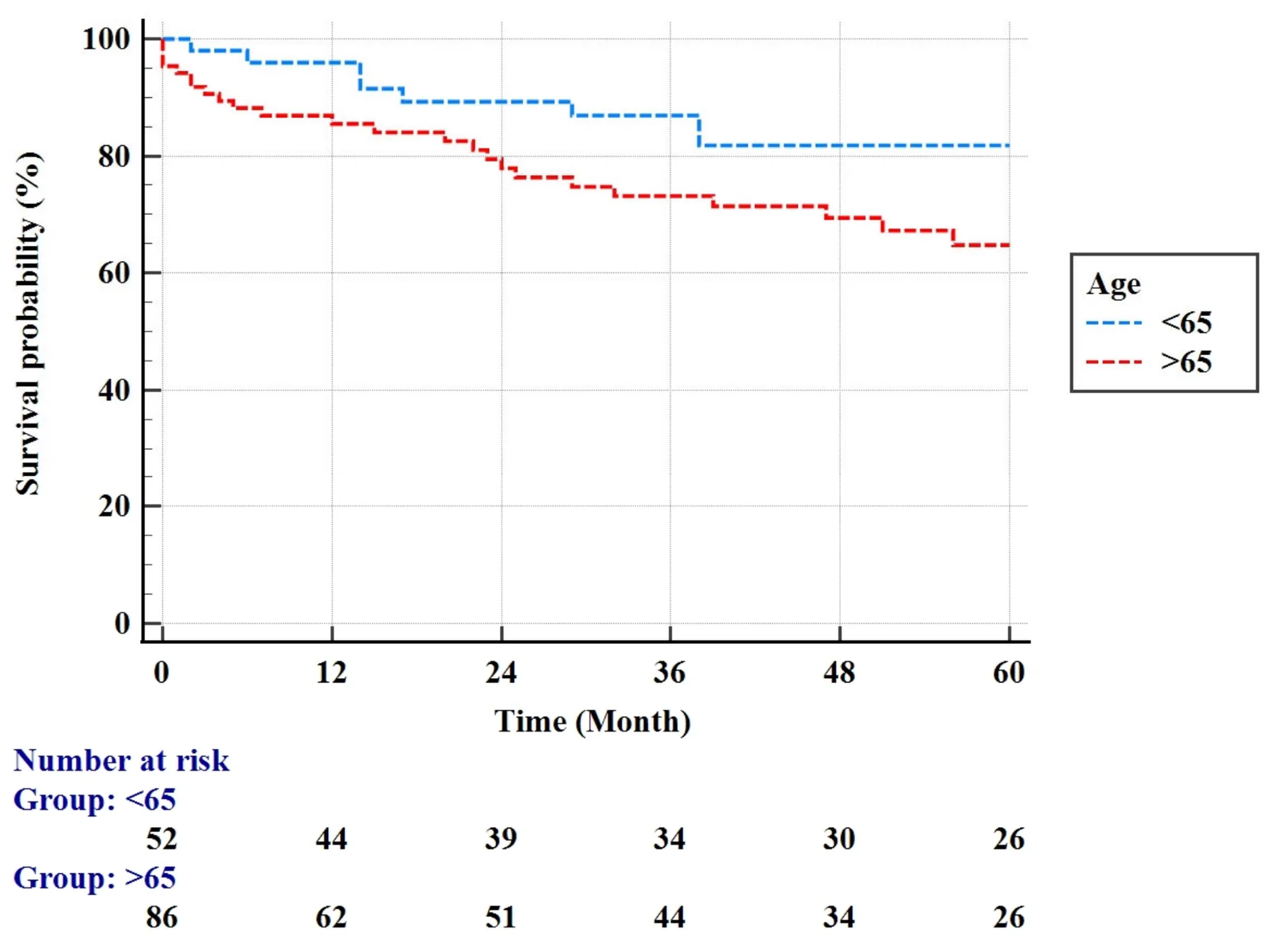
Kaplan–Meier curves of cancer-specific survival stratified by age group (<65 vs. ≥65 years).

**Figure 3:**
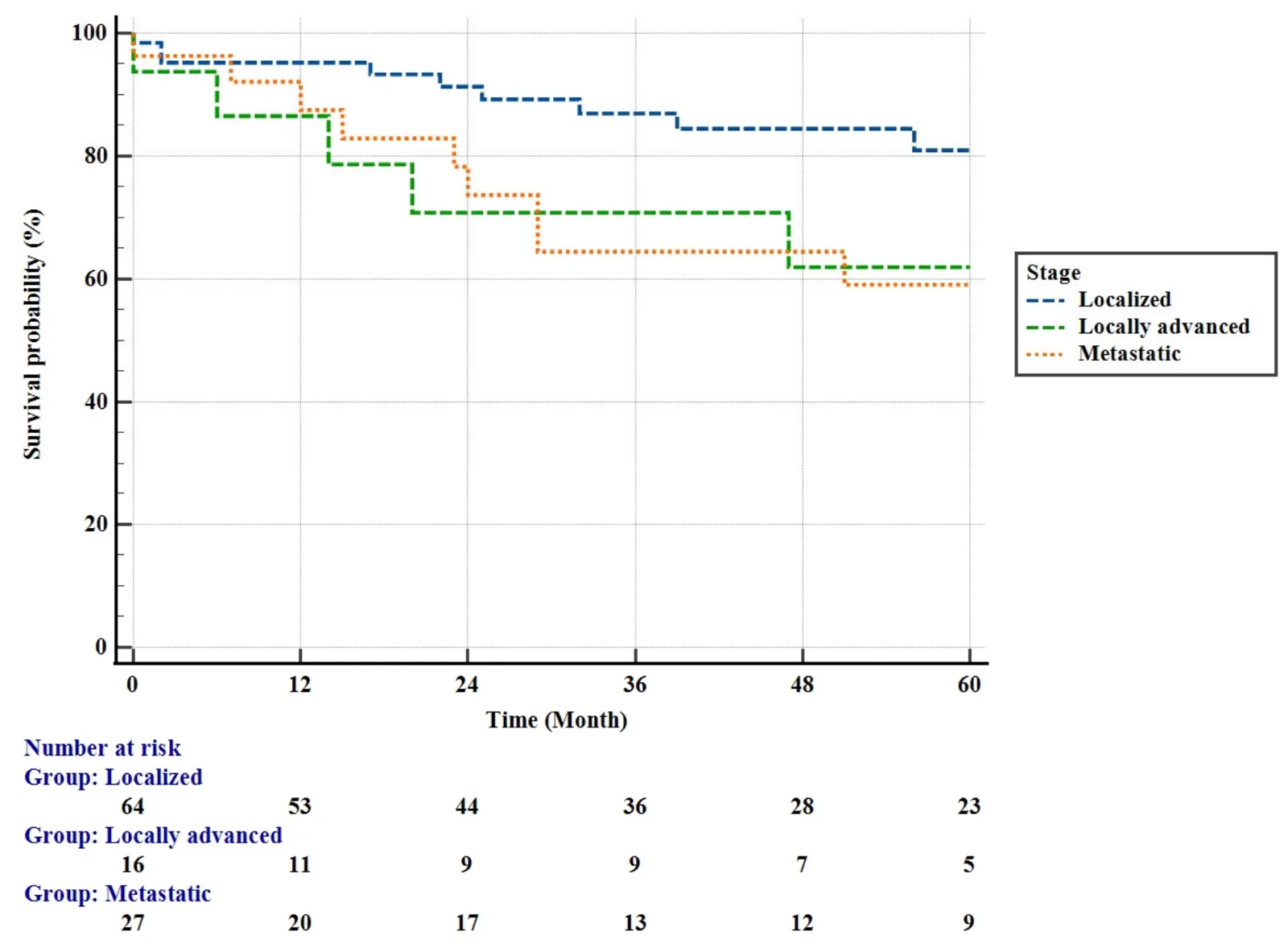
Kaplan–Meier curves of cancer-specific survival stratified by stage group.

CSS differed by age group. Patients aged <65 years had higher CSS than those aged ≥65 years during follow-up (Figure 2). CSS also differed by stage group, with the highest survival in localized disease, followed by locally advanced and metastatic disease (Figure 3). Patients recorded as receiving chemotherapy or radiotherapy had higher unadjusted CSS curves than those not recorded as receiving these treatments. Overall, 62 patients (44.9%) were recorded as receiving chemotherapy, and 43 patients (31.2%) were recorded as receiving radiotherapy (Table 1).

In Cox regression analysis, age and histologic subtype were associated with CSS. Patients aged ≥65 years had worse CSS than younger patients in both univariable and multivariable analyses. Compared with DLBCL, MALT lymphoma and follicular lymphoma were associated with better CSS. Ann Arbor stage IV was associated with worse CSS in univariable analysis; stage was not included in the multivariable model. Radiotherapy and chemotherapy were associated with better CSS in univariable analysis, but these associations were not statistically significant after adjustment. Primary breast surgery was not associated with CSS in either univariable or multivariable analysis (Table 2).

## 4. Discussion

This population-based study examined the clinicopathological characteristics and cancer-specific survival (CSS) of men with breast lymphoma recorded in SEER. Male breast lymphoma is an exceptionally rare entity, and most available data come from case reports, female-predominant series, or mixed cohorts in which only a small number of men were included [2,3,4]. In the present cohort, most patients were older, and DLBCL was the most common histological subtype. This distribution is similar to that reported in previous registry studies and published male PBL cases [3,5]. Zhang et al. previously reported a SEER-based analysis of 122 male patients diagnosed between 2000 and 2019 [3]. By extending the study period to 2021 and including 138 patients, our analysis provides a more updated population-based overview of this rare disease. We also clarified that SEER primary-site coding identifies lymphoma recorded as the primary breast site, but cannot confirm strict clinical PBL criteria for every patient.

Older age was associated with poorer CSS. This finding is clinically plausible, as age is a well-known prognostic factor in DLBCL and other lymphomas [13]. In older patients, survival may be influenced not only by lymphoma biology, but also by frailty, comorbidities, treatment tolerance, and the ability to complete planned therapy. These factors are not captured in SEER. Therefore, the worse outcome observed in patients aged ≥65 years may partly reflect lower treatment intensity or shorter treatment duration [14]. This finding supports the need for careful patient assessment in older individuals, including evaluation of fitness, supportive care requirements, and treatment feasibility.

In this study, chemotherapy was not independently associated with CSS after adjustment. This result should be understood within the limitations of registry data. Chemotherapy was recorded in fewer than half of the patients, and only 41 cancer-specific death events were observed. SEER does not provide information on chemotherapy regimen, rituximab use, dose intensity, number of cycles, treatment completion, response, or the reason why chemotherapy was given or withheld. Therefore, the absence of an independent association in this analysis should not be interpreted as evidence against chemotherapy. In routine lymphoma practice, systemic therapy remains a key component of treatment for patients with aggressive histology or advanced disease who are suitable candidates. Because chemotherapy is more often used in patients with higher-risk disease, crude survival comparisons may be biased. Previous studies in the rituximab era have reported favorable outcomes with anthracycline-based chemoimmunotherapy in primary breast DLBCL, and radiotherapy may have a role in selected localized cases [6,7,8]. This is also in keeping with the treatment approach for limited-stage DLBCL, where systemic therapy is central and radiotherapy is considered according to patient and disease factors [15]. For this reason, treatment-related findings in the present study should be regarded as associations rather than direct estimates of treatment benefit.

The management of extranodal lymphoma has changed considerably with advances in immunochemotherapy and lymphoma classification [16]. In aggressive B-cell lymphomas, anti-CD20-based therapy has improved outcomes and has shifted clinical decision-making from an anatomic-site-based approach toward one that incorporates histology, disease biology, stage, and patient fitness [17]. Current guidelines also recommend treatment according to lymphoma subtype, disease extent, and patient-related factors rather than breast-directed surgery alone [18]. The lack of survival benefit associated with primary breast surgery in our analysis is compatible with this modern treatment concept, although the surgical variable in SEER should be interpreted carefully because the database does not always distinguish diagnostic procedures from therapeutic surgery.

Stage-stratified survival showed a gradual decrease from localized to metastatic disease, supporting the clinical relevance of disease extent at diagnosis. The Lugano classification recommends PET/CT-based staging and standardized response assessment for aggressive lymphomas [19]. However, SEER stage information was incomplete, and registry-derived stage groups do not fully correspond to Lugano staging. Despite this limitation, the observed survival difference across stage groups suggests that stage remains important in male breast lymphoma. Histological subtype was also strongly associated with outcome. Patients with MALT lymphoma and follicular lymphoma had better CSS than those with DLBCL, which reflects the generally more indolent clinical course of these lymphomas [5,20]. Breast MALT lymphoma may achieve durable disease control with radiotherapy or systemic treatment when needed, whereas DLBCL usually requires systemic chemoimmunotherapy [5,18,20]. Because male breast lymphoma is rarely encountered in routine practice, pooled data from multiple centers would be valuable for clarifying prognosis, treatment selection, and long-term outcomes in this patient group.

## 5. Study Limitations

This study has several limitations. First, its registry-based design limits the clinical detail available for analysis. SEER does not include performance status, LDH level, International Prognostic Index components, chemotherapy regimen, rituximab use, radiotherapy dose or field, CNS prophylaxis, treatment response, toxicity, or relapse pattern. Therefore, treatment-related results cannot be interpreted as causal effects. Stage and B-symptom data were also incomplete, and SEER stage groups may not fully reflect PET/CT-based Lugano staging. In addition, SEER cause-of-death classification may be imperfect, particularly in an older population, and this may affect CSS estimates.

Another limitation is the small number of cancer-specific death events. Only 41 events were observed, which limited the number of variables that could be included in the multivariable model. Therefore, the multivariable analysis was limited to variables considered suitable for inclusion. Stage was not included in the final model because of incomplete stage information, its relationship with treatment type, and the risk of model instability. In addition, chemotherapy is usually given to patients with aggressive histology or more advanced disease, which may affect unadjusted survival comparisons. Although adjustment reduced this imbalance, residual confounding remains possible.

Finally, the rarity of male breast lymphoma limits detailed subgroup analyses and precludes firm treatment recommendations. Despite these limitations, this study provides useful population-based information on a rare disease that is difficult to evaluate in single-center series.

## 6. Conclusions

In this SEER-based study of 138 men with breast lymphoma diagnosed between 2000 and 2021, older age and aggressive histology were independently associated with poorer cancer-specific survival. Stage, chemotherapy, and radiotherapy were associated with survival mainly in univariable analyses. These findings require careful interpretation because of missing data, treatment selection, cause-of-death coding limitations, and the lack of regimen-level information in SEER. This study adds value by providing one of the largest and most updated male-only SEER analyses of this rare disease. Further pooled clinical data are needed to better define prognosis and support treatment decisions in this rare patient group.

## Figures and Tables

**Table 1:** Baseline Characteristics of the Patients with Male Breast Lymphoma (SEER, 2000–2021).

Characteristic	*n*: 138	%
**Age** <65 ≥65	52 86	37.7 62.3
**Race** White Black Asian/Pacific	122 9 7	88.4 6.5 5.1
**Origin** Non-Hispanic-Latino Hispanic-Latino	114 24	82.6 17.4
**Tumor laterality** Right breast Left breast Bilateral Unknown	67 65 5 1	48.6 47.1 3.6 0.7
**Histological subtype** DLBCL MALT lymphoma Follicular lymphoma Other	54 33 24 27	39.1 23.9 17.4 19.6
**Ann Arbor stage** I II III IV Unknown	39 17 5 19 58	28.3 12.3 3.6 13.8 42.0
**B symptoms** Yes No Unknown	4 56 78	2.9 40.6 56.5
**Breast surgery** Yes No Unknown	41 94 3	29.7 68.1 2.2
**Radiotherapy** Yes No	43 95	31.2 68.8
**Chemotherapy** Yes No	62 76	44.9 55.1

**Table 2:** Univariable and Multivariable Cox Regression Analysis of Factors Associated with Cancer-specific survival in Men with Breast Lymphoma Included in the SEER Database Between 2000 and 2021.

	Univariable Analysis		Multivariable Analysis	
	Hazard Ratio for CSS (95% CI)	*p*-Value	Hazard Ratio for CSS (95% CI)	*p*-Value
**Age** *<65 years* *≥65 years*	Reference 3.67 (1.67–8.06)	0.001	Reference 4.13 (1.83–9.33)	0.001
**Race** *White* *Black* *Asian/Pacific*	Reference 1.59 (0.56–4.49) 0.55 (0.07–4.00)	0.375 0.551	—	—
**Origin** *Non-Hispanic* *Hispanic*	Reference 0.94 (0.41–2.12)	0.888	—	—
**Laterality** *Right* *Left* *Bilateral*	Reference 1.16 (0.62–2.17) 0.57 (0.07–4.33)	0.627 0.595	—	—
**Histological subtype** *DLBCL* *MALT lymphoma* *Follicular lymphoma* *Other*	Reference 0.30 (0.13–0.71) 0.12 (0.02–0.52) 0.41 (0.18–0.93)	0.006 0.005 0.033	Reference 0.36 (0.14–0.94) 0.14 (0.03–0.68) 0.43 (0.18–1.03)	0.037 0.015 0.061
**Ann Arbor stage** *I* *II* *III* *IV*	Reference 1.26 (0.48–3.34) 1.95 (0.55–6.88) 2.95 (1.33–6.53)	0.629 0.295 0.007	—	—
**B symptoms** *Present* *Absent*	Reference 1.34 (0.17–10.27)	0.778	—	—
**Primary Breast Surgery** *No/Unknown* *Yes*	Reference 1.21 (0.63–2.33)	0.556	Reference 0.84 (0.43–1.67)	0.637
**Radiotherapy** *Yes* *No/Unknown*	Reference 2.19 (1.04–4.62)	0.038	Reference 1.72 (0.80–3.72)	0.164
**Chemotherapy** *Yes* *No/Unknown*	Reference 0.46 (0.24–0.88)	0.020	Reference 0.66 (0.31–1.39)	0.276

Multivariable analysis model *p*-value < 0.001. Abbreviations: CI, confidence interval; HR, hazard ratio; CSS, cancer specific survival; DLBCL, diffuse large B-cell lymphoma; MALT, mucosa-associated lymphoid tissue.

## Data Availability

The datasets generated and/or analyzed during the current study are available in the Surveillance, Epidemiology, and End Results (SEER) Program repository, https://seer.cancer.gov/data-software/ (accessed on 15 June 2025).
